# Wearing of Cloth or Disposable Surgical Face Masks has no Effect on Vigorous Exercise Performance in Healthy Individuals

**DOI:** 10.3390/ijerph17218110

**Published:** 2020-11-03

**Authors:** Keely Shaw, Scotty Butcher, Jongbum Ko, Gordon A. Zello, Philip D. Chilibeck

**Affiliations:** 1 College of Kinesiology, University of Saskatchewan, Saskatoon, SK S7N 5B2, Canada; keely.shaw@usask.ca (K.S.); jongbum.ko@usask.ca (J.K.); 2 School of Rehabilitation Science, University of Saskatchewan, Saskatoon, SK S7N 2Z4, Canada; Scotty.butcher@usask.ca; 3 College of Pharmacy and Nutrition, University of Saskatchewan, Saskatoon, SK S7N 5E5, Canada; Gordon.zello@usask.ca

**Keywords:** physical activity, coronavirus, maximal oxygen uptake, pulse oximetry, near-infrared spectroscopy, COVID-19, pandemic

## Abstract

Wearing face masks is recommended for the prevention of contracting or exposing others to cardiorespiratory infections, such as COVID-19. Controversy exists on whether wearing face masks during vigorous exercise affects performance. We used a randomized, counterbalanced cross-over design to evaluate the effects of wearing a surgical mask, a cloth mask, or no mask in 14 participants (7 men and 7 women; 28.2 ± 8.7 y) during a cycle ergometry test to exhaustion. Arterial oxygen saturation (pulse oximetry) and tissue oxygenation index (indicator of hemoglobin saturation/desaturation) at vastus lateralis (near-infrared spectroscopy) were assessed throughout the exercise tests. Wearing face masks had no effect on performance (time to exhaustion (mean ± SD): no mask 622 ± 141 s, surgical mask 657 ± 158 s, cloth mask 637 ± 153 s (*p* = 0.20); peak power: no mask 234 ± 56 W, surgical mask 241 ± 57 W, cloth mask 241 ± 51 W (*p* = 0.49)). When expressed relative to peak exercise performance, no differences were evident between wearing or not wearing a mask for arterial oxygen saturation, tissue oxygenation index, rating of perceived exertion, or heart rate at any time during the exercise tests. Wearing a face mask during vigorous exercise had no discernable detrimental effect on blood or muscle oxygenation, and exercise performance in young, healthy participants (ClinicalTrials.gov, NCT04557605).

## 1. Introduction

The coronavirus-19 (COVID-19) pandemic has created serious challenges to global health, for which face mask use is recommended as a mitigation strategy [1]. Evidence exists that the use of face masks prevents contracting and transmitting COVID-19 [2,3,4]. Surgical face masks and N-95 face masks are effective, but cloth face masks have been recommended for use by the general public in order to preserve the supply of surgical or N-95 masks for medical personnel [1]. Social distancing is recommended in the absence of face masks and a “two-meter rule” is generally recommended; however, this distance might need to be increased during vigorous exercise, where respiratory droplets may travel further during high rates of forceful respiration [5]. Wearing face masks during vigorous exercise might, therefore, be important for the prevention of spread of infectious respiratory droplets; however, the ability to exercise vigorously while wearing a face mask is a concern [6]. One hypothesis is that oxygen uptake will be compromised and that trapping of air in the face mask will increase rebreathing of carbon dioxide, leading to hypercapnic hypoxia, where an increase in arterial carbon dioxide displaces oxygen from hemoglobin [6]. Another hypothesis is that face masks will increase resistance to inspiration and respiration, thus increasing the work of breathing [6]. Exercise is effective for the prevention of obesity, diabetes, and hypertension [7,8,9], all of which are leading risk factors for complications if one contracts COVID-19 [10,11,12]. It is therefore important to determine if vigorous exercise is compromised while wearing face masks in order to make exercise prescription recommendations.

There are mixed results as to whether wearing a face mask impairs exercise performance. Participants had higher ratings of perceived exertion and slightly higher heart rate while walking at 4 km/h for 6 min on a graded treadmill (10% grade) while wearing a surgical face mask compared to when not wearing a face mask, but exercise performance per se was not assessed [13]. Maximum power reached during a progressive cycle ergometer test (where work rate started at 50 W and was increased 50 W every 3 min until exhaustion) was reduced while wearing a face mask [14]. A rubber face mask was used to evaluate gas exchange (i.e., this was placed over a surgical or N-95 mask). This effectively sealed the surgical or N-95 mask to the face, which may have made exercise more difficult with the face mask, potentially compromising external validity (i.e., the applicability of the results to a real-world setting). An additional study, again using a progressive cycle ergometer test (starting at 25 W and increasing by 25 W every three minutes until exhaustion) found no difference in time to exhaustion while wearing surgical or N-95 masks compared to a no-mask condition [15]. Nasal prongs were inserted up the nostrils to evaluate respiratory rate and end-tidal carbon dioxide during exercise. Again, this may have compromised external validity because this may interfere with the face mask.

The purpose of our study was to evaluate whether exercise performance during a progressive cycle ergometer test is impaired by wearing face masks, where no apparatus was applied to the face to assess breathing, thereby not compromising external validity. We chose to evaluate disposable surgical and non-disposable cloth face masks because these are effective for reducing the transmission of exhaled droplets [16]. They are also commercially available and are therefore commonly worn by the general public. We also evaluated both blood and muscle oxygen levels (by pulse oximetry and near-infrared spectroscopy, respectively). Given that face masks are proposed to inhibit oxygen uptake, increase carbon dioxide rebreathing, and increase work of breathing due to increased resistance to inspiration and expiration [6], our working hypothesis was that wearing a face mask during exercise would reduce blood and muscle oxygenation resulting in reduced exercise performance.

## 2. Materials and Methods

This study used a randomized counterbalanced cross-over design where 14 participants (7 men (mean ± SD): age 26.1 ± 5.8 years, mass 86 ± 12 kg, height 180 ± 5 cm, and 7 women: age 30.3 ± 10.9 years, mass 77 ± 17 kg, height 167 ± 5 cm), who participated in 288 ± 197 min of moderate to vigorous physical activity per week, were randomized to perform a progressive cycle ergometer exercise test to exhaustion while wearing a cloth face mask, a disposable surgical face mask, or no mask on three separate occasions, with at least 48 h separating conditions. Each session took place at approximately the same time of the day (±1.5 h). Randomization was performed using a computerized random number generator. The research assistant who generated the allocation schedule concealed the schedule from another research assistant who enrolled participants (i.e., this research assistant contacted the research assistant with the allocation schedule by email for the next condition each time a participant was brought in for testing).

Participants were healthy and were required to fill out the “Get Active Questionnaire” (https://store.csep.ca/pages/getactivequestionnaire) to screen for contraindications to exercise testing before being enrolled into the study. This questionnaire also allowed an assessment of weekly moderate and vigorous activity, as it has a question on the number of times moderate/vigorous activity is performed per week and typical durations of exercise sessions. This study was registered at ClinicalTrials.gov (NCT04557605). The study was approved by the University of Saskatchewan Biomedical Ethics Review Board and all participants signed a consent form prior to participating.

Sample size was calculated based on day-to-day variation (i.e., reproducibility) for peak power output generated during the progressive cycle ergometer test, and the expected decrease during conditions of mild hypoxia. The day-to-day variation, as a coefficient of variation for this test, was previously determined in nine participants as 3.8% [17]. The expected decrease in peak power output with mild hypoxia is about 5% [18]. With this coefficient of variation and expected change, we required 13 participants with an alpha value of 0.05 and power of 90% [19].

The disposable surgical mask was a 3-ply ear-loop facemask (CRC-Elm 5, Hawketree Solutions, Ottawa, ON, Canada). The cloth mask (Washable 3D Face Mask, TriMax Sports Inc., Vancouver, BC, Canada) was a 3-layer ear-loop face mask with layers composed of bamboo charcoal cloth, non-woven fabric, and Lycra (going from inner to outer layer), and was selected to represent face masks that are typically used by the general population [1,3].

The progressive exercise test was performed on a Monark Ergomedic 828E cycling ergometer (Vansboro, Sweden). The cycling protocol initially involved resting for 5 min and then performing a 5-min warm-up at a self-selected resistance, which was recorded and used on subsequent visits (face masks were worn during rest and warm-up if the participant was doing one of the face mask conditions). The starting power output on the ergometer ranged from 35 to 100 W depending on the fitness level and was increased 35 W every 2 min until volitional fatigue, with participants cycling at 70–75 revolutions per minute [17]. Starting load (power output) for each participant was recorded and replicated on subsequent conditions. Heart rate (Polar Electro, New Hyde Park, NY, USA), arterial blood oxygen saturation monitored with a pulse oximeter (Nellcor Oximax NPB40MAX, Medtronic Canada, Brampton, ON, Canada), and rating of perceived exertion (10-point scale) were recorded every 30 s. Continuous-wave near-infrared spectroscopy (NIRS; NIRO-200NX, Hamamatsu Photonics, Hamamatsu City, Shizuoka Pref., Japan) was used to measure the oxyhemoglobin content of the right vastus lateralis, as previously described [20], with values averaged every 20 s. NIRS probe placement was one-third of the distance from patella to inguinal line, with the lateral probe (approximately 4 cm) outlined on the skin with a marker to ensure consistent placement. Tissue oxygenation index, which represents absolute oxygen saturation of tissue hemoglobin expressed as a percentage, was determined by oxyhemoglobin divided by total hemoglobin.

During the exercise tests, participants were blinded to the time elapsed so that knowledge of their exercise time would not influence subsequent tests. Data were entered into a spreadsheet with conditions coded so that statistical analyses could also be performed in a blinded manner.

To control for effects of diet, previous physical activity, and sleep, participants were required to fill out a food diary and record their physical activity and sleep duration 24 h before the first exercise test. They were given a photocopy of this diary and asked to replicate as close as possible their diet, physical activity, and sleep before subsequent testing occasions.

For statistical analyses, data were analyzed using Statistica 5.0 (Chicago IL, USA). Time to exhaustion and peak power output during the tests were assessed with a one-factor repeated-measures ANOVA with “condition” (cloth face mask vs. surgical face mask vs. no mask) as the independent variable. Blood oxygen saturation, muscle tissue oxygenation index, heart rate, and rating of perceived exertion between conditions were assessed in several ways since duration of exercise tests varied across conditions. A one-factor repeated-measures ANOVA was used to compare conditions at the end of the exercise test (i.e., at exhaustion). Data were also expressed relative to peak power (i.e., percentage of peak power) and analyzed by a two-factor (i.e., percentage of peak power × condition) repeated-measures ANOVA. As a secondary analysis, “sex” was added as a between-group factor to all analyses. Bonferroni post-hoc tests were used to compare pairs of means if there were condition main effects, or condition × % peak power, condition × sex, or condition × % peak power × sex interactions. All results were expressed as mean ± SD and significance was accepted at *p* ≤ 0.05.

## 3. Results

**Figure 1:**
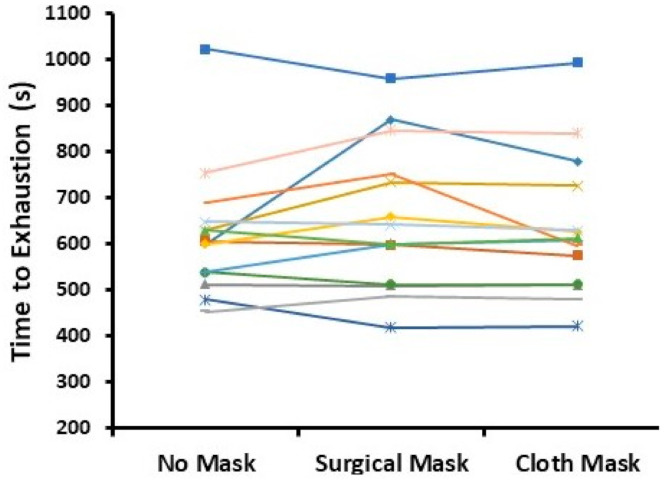
Time to exhaustion during the exercise test for individual participants across conditions. There were no statistical differences between conditions (*p* = 0.20).

Time to exhaustion during the exercise test was not different for face mask compared to no face mask conditions (622 ± 141, 657 ± 158, and 637 ± 153 s for no mask, surgical mask, and cloth mask conditions, respectively; Figure 1; *p* = 0.20; power = 33%). Further, there were no differences between sexes and no condition × sex interaction. 

No differences were found between conditions for peak power reached during the exercise test (*p* = 0.49; power = 16%). Peak power was 234 ± 56, 241 ± 57, and 241 ± 51 W for no mask, surgical mask, and cloth mask conditions, respectively. Females had a lower peak power during the exercise test compared with males (*p* = 0.034), but there was no condition × sex interaction.

**Figure 2:**
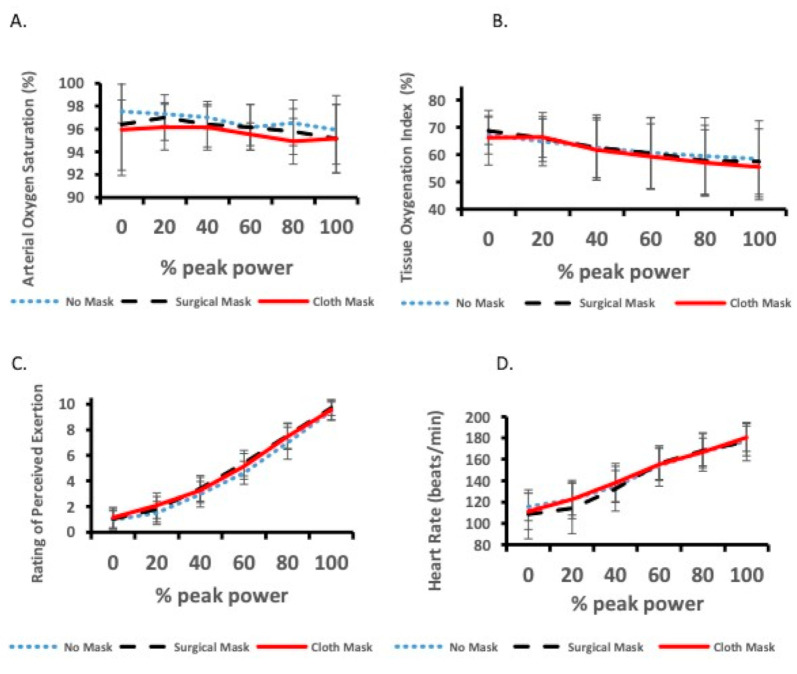
(**A**) Arterial oxygen saturation, (**B**) muscle tissue oxygenation index, (**C**) rating of perceived exertion, and (**D**) heart rate expressed as percentage of peak power during the exercise test. All values are mean ± SD.

Arterial oxygenation data in one male participant could not be collected due to equipment error. For arterial oxygen saturation, there was no difference at the end of exercise between face mask and no mask conditions (Table 1; *p* = 0.34). When values were expressed relative to peak power throughout the exercise test, there were no main effects due to condition (*p* = 0.50) or % peak power (*p* = 0.35), and no interaction between condition and % peak power (*p* = 0.89) (Figure 2a). For all analyses, no differences between the sexes and no sex × condition or sex × condition × % peak power interactions were present.

For NIRS-derived muscle tissue oxygenation index, face mask and no face mask conditions were not different at the end of exercise (Table 1; *p* = 0.55). There was a sex main effect with males, which was lower than that of females at the end of exercise (50 ± 10% vs. 66 ± 10%; *p* = 0.013); however, there were no sex × condition interactions. When values were expressed relative to peak power throughout the exercise test, there was a significant condition × % peak power interaction (*p* = 0.047); however, differences between conditions were not evident from Bonferroni post-hoc analyses on this interaction. For each condition, tissue oxygenation index significantly dropped from 20% peak power onwards (all *p* < 0.01); however, there were no differences between conditions at any relative power outputs (Figure 2b). Again, there was a sex main effect with males, which was lower than that of females (56 ± 8% vs. 68 ± 8%), but no sex × condition × % power interaction. No difference was shown for decrease in tissue oxygenation index throughout the exercise test between conditions (20 ± 16%, 20 ± 16%, and 20 ± 14% decrease for no mask, surgical mask, and cloth mask, respectively; *p* = 0.96). Males had a greater decrease in tissue oxygenation index compared with females (30 ± 11% vs. 10 ± 11%; *p* = 0.005).

For rating of perceived exertion, face mask and no face mask conditions were not different at the end of exercise (Table 1; *p* = 0.47). When values were expressed relative to peak power throughout the exercise test, there were no main effects due to condition (*p* = 0.12) and no interaction between condition and % peak power (*p* = 0.39). As expected, there was a main effect for % peak power (*p* < 0.01) with rating of perceived exertion increasing throughout the exercise test (Figure 2c). For all analyses, no differences between the sexes and no sex × condition or sex × condition × % peak power interactions were evident.

Heart rate data for one male participant were excluded because of equipment error. For heart rate, face mask and no face mask conditions were not different at the end of exercise (Table 1; *p* = 0.41). When values were expressed relative to peak power throughout the exercise test, there were no main effects due to condition (*p* = 0.71) and no interaction between condition and % peak power (*p* = 0.44). As expected, there was a main effect for % peak power (*p* < 0.01) with heart rate increasing throughout the exercise test (Figure 2d). For all analyses, no differences between the sexes and no sex × condition or sex × condition × % peak power interactions were evident.

## 4. Discussion

The main finding from this study was that exercise performance, measured as either time to exhaustion or peak power output during an incremental cycle ergometer test, was not affected by wearing either a disposable surgical face mask or a non-disposable cloth face mask. Additionally, face mask and no face mask conditions were not different throughout the exercise test when oxygen saturation was assessed relative to peak power output. Our findings are of importance because they indicate that people can wear face masks during intense exercise with no detrimental effects on performance and minimal impact on blood and muscle oxygenation. This is important when fitness centers open up during COVID-19 since respiratory droplets may be propelled further with heavy breathing during vigorous exercise [5] and because of reports of COVID-19 clusters in crowded enclosed exercise facilities [21]. Our findings indicate that face masks can be worn during exercise without affecting exercise performance or blood and muscle oxygenation.

Our findings were contrary to our hypothesis that exercise performance would be negatively affected by wearing a face mask. This was based on the hypothesis proposed by Chandrasekaran et al. [6] that wearing a face mask during exercise would increase rebreathing of carbon dioxide or that oxygen consumption would be compromised, both of which would lead to lower arterial oxygen saturation of hemoglobin. Chandrasekaran et al. [6] also proposed that face masks might provide resistance to breathing, making work of breathing more difficult. Some evidence from previous studies supported these physiological effects. For example, at rest, wearing a surgical mask reduced peak expiratory flow, force vital capacity, and forced expiratory volume measured over one second [14]. Wearing a surgical face mask also reduced peak ventilation during a progressive cycle ergometry test; however, wearing a spirometry mask (i.e., to evaluate gas exchange) over the surgical mask may have essentially sealed the surgical mask to the face, reducing the external validity of measurements in this study. Epstein et al. [15] measured an increase in end-tidal carbon dioxide while wearing a surgical face mask at exhaustion during a progressive cycle ergometer test; however, this did not affect arterial oxygen saturation during exercise and had no detrimental effect on performance in their study. Our study indicated that none of these potential negative effects of wearing a face mask had significant effects on performance.

Our results for pulse oximetry were in agreement with other studies assessing face masks during progressive-intensity cycle ergometry tests. Epstein et al. [15] observed no decrease in arterial oxygen saturation expressed as a percentage of peak power output and Fikenzer et al. [14] observed no differences in partial pressure of oxygen, measured at exhaustion in earlobe blood samples. Our study was the first to evaluate muscle oxygen levels (i.e., tissue oxygenation index). Although a significant condition × % power interaction for tissue oxygenation index was shown, the Bonferroni post-hoc analyses on this interaction indicated no significant differences between face mask and no face mask conditions at any % power during the test. In addition, when we assessed percent decline in tissue oxygenation index during the exercise tests, there were no differences between face mask condition and the no face mask condition. Overall, our study showed no substantial effects of wearing a face mask during exercise on either blood or muscle oxygenation.

We found no differences in ratings of perceived exertion or heart rate between conditions throughout the exercise test. This was in agreement with one other study comparing surgical face masks to no face masks during a progressive-intensity cycle ergometry test [15], but was in contrast to a study that found significantly elevated heart rate and rating of perceived exertion while treadmill walking at 4 km/h on a steep grade for 6 min (i.e., to simulate hiking in the mountains) while wearing a surgical face mask [13]. Differences between studies might be due to differences in exercise modes, methods of exercise testing (i.e., maximal vs. submaximal), or participant fitness levels. Our findings of no difference between face mask and no face mask conditions for ratings of perceived exertion or heart rate during exercise supported our results for performance and blood and muscle oxygenation, where no substantial differences were detected between conditions.

Our study showed no differences in performance, as assessed by time to exhaustion or peak power output while wearing cloth or surgical face masks during a progressive intensity exercise test. Previous studies evaluating surgical masks during the same type of exercise test provided contrasting results with one stating that exercise performance was impaired while wearing a surgical mask [14] and the other finding no impairment with a surgical mask [15]. Of note is that upon close inspection of the study by Fikenzer et al. [14], the difference in performance between surgical-mask vs. no-mask conditions was only 3% (i.e., peak power of 269 W vs. 277 W) and this difference actually failed to reach statistical significance (*p* = 0.07) (the abstract for the study, however, concluded that “cardiopulmonary exercise capacity and comfort are reduced by surgical masks”). Both of these studies used measurement devices during their exercise tests to assess respiration or expired gases (i.e., either a spirometry mask that was worn over the surgical mask or nasal prongs that were inserted into the nostrils). Although this provided valuable data on respiration and gas exchange, we believe that this compromised the evaluation of exercise performance while wearing a face mask as both did not present real-life conditions (i.e., compromised external validity). Wearing a spirometry mask over the surgical mask seals or tightens the surgical mask to the face, potentially making breathing more difficult because gases would not be able to escape from the sides of the mask. Wearing nasal prongs in the nostrils during exercise may also affect the wearing of face masks. Although we did not have an assessment of respiratory gases in our study, we think that the lack of measurement devices around the nose or mouth area increased the external validity of our exercise performance measures (i.e., time to exhaustion and peak power). Another strength of our study was the inclusion of both males and females, which has been done only in one other exercise and face mask study [13]. The recommendations for safe use of face masks during exercise from our study can therefore be applied to healthy males and females who choose to wear face masks while exercising.

Our study had several limitations. We evaluated a 3-layer cloth face mask, rather than a single-layer cloth mask. We chose this mask as it was one of the “higher end” masks on the market. Results using a single-layer cloth mask may differ. We evaluated the wearing of masks during a progressive exercise test to exhaustion. This is not typical of a regular aerobic exercise training session, which most likely involves 30 min or more at a submaximal intensity. A final limitation was that the tissue oxygenation index measures had a large standard deviation, which made it difficult to detect differences between conditions despite a significant condition × % peak power interaction.

## 5. Conclusions

In summary, our study found no detrimental effect of wearing either a non-disposable cloth or disposal surgical face mask while exercising vigorously on exercise performance. For healthy, active people, wearing a face mask during vigorous exercise has minimal effect on arterial or muscle oxygen levels and no effects on exercise performance. This has practical significance, especially when exercising in settings where individuals might be vulnerable to contracting COVID-19, such as enclosed gyms [21], given that exercise should be encouraged for everyone during COVID-19 to reduce many of the risk factors (i.e., obesity, diabetes, and high blood pressure) that are associated with the worst COVID-19 outcomes [10,11,12].

## Figures and Tables

**Table 1:** Comparison between conditions at the end of the exercise test.

	No Mask	Surgical Mask	Cloth Mask
Blood oxygen saturation (%)	96 ± 4	96 ± 3	95 ± 3
Tissue oxygenation index (%)	58 ± 14	57 ± 12	58 ± 12
Rating of perceived exertion	9.9 ± 0.5	9.9 ± 0.4	9.7 ± 0.6
Heart rate (beats per minute)	179 ± 16	179 ± 19	182 ± 12

All values are mean ± SD.
